# Supplementary material for: WordCommentsAnalyzer: A windows software tool for qualitative research
Source: F1000Res. 2018 Sep 4;7:536. Originally published 2018 May 3. [Version 2] doi: 10.12688/f1000research.14819.2 (PMC6137411; doi:10.12688/f1000research.14819.2)
Supplement: Supplementary file 3 [file f1000research-7-17614-s0002.tgz › b2a79496-4ada-47c4-9682-a99ce867836d.zip!/Eight Iranian Health Journals/Journal of Knowledge and Health_5.docx]

© Journal of Knowledge and Health. This work is licensed under CC BY 4.0 (https://creativecommons.org/licenses/by/4.0/).

Volume & Issue: دوره 12، شماره 1: 1396

Title: علل تقاضای القایی پزشکان در ارايه خدمات به بیمه‌شدگان بیمه سلامت: مطالعه مورد استان اصفهان، 1392

http://knh.shmu.ac.ir/index.php/site/article/view/1298

مقدمه: تقاضای القایی یکی از چالش‌های سلامت کشور ما مي‌باشد. تقاضای القایی در مواردی که هزینه‌های توسط بیمه پرداخت مي‌گردد، مي‌تواند سبب هدر رفتن منابع نظام سلامت گردد. مواد و روش‌ها: مطالعه توصیفی- تحلیلی حاضر به‌منظور بررسی علل تقاضای القایی به کمک اطلاعات جمع‌آوری شده از پزشکان که خدمات سطح یک را در سال 92 به بیمه‌شدگان سلامت استان اصفهان ارايه دادند، انجام شد. از مدل لوجیت و نرم‌افزار ایویوز برای تحلیل یافته‌ها استفاده شد. در مدل لوجیت از دو متغیر وابسته هزینه کل و زمان کل استفاده شد. اثر نهایی متغیر‌های توضیحی بر این دو متغیر به‌عنوان تأثير نهایی بر تقاضای القایی مورد بررسی قرار گرفت. نتایج: در مدل تصریح شده هزینه کل، نوع قرارداد پزشک (0195/0=P)، سن پزشک (0474/0=P) و ویزیت بیمار زن (001/0>P) از لحاظ آماری معنی‌دار شدند. در مدل تصریح شده زمان کل، نتایج نوع قرار داد پزشک (0008/0=P)، ویزیت بیمار بالای 60 سال (0144/0=P)، ویزیت بیمار زیر 5 سال (0001/0=P) و تعداد ارجاع (019/0=P) از لحاظ آماری معنی‌دار شدند. اثر نهایی به‌دست آمده در هزینه کل نوع قرارداد پزشک 11% به‌دست آمد و از نظر زمان کل نیز نوع قرارداد پزشک با بیشترین اثر 19% به‌دست آمد. نتیجه‌گیری: با‌توجه به نتایج این پژوهش متولیان سیاستگذاری سلامت، باید با اتخاذ روش علمی مبتنی‌بر خرید خدمت توسط سازمان‌های بیمه‌گر، تصویب استانداردها و قوانین و تعیین چهارچوب درمان منطقی و علمی راه را بر عرضه خدمات غیرضروری بسته و منابع سلامت را به‌صورت بهینه تخصیص دهند.

Title: بررسی ارتباط افسردگی و کیفیت زندگی در سالمندان مقیم و غیر‌مقیم سرای سالمندان شهرستان شاهرود

http://knh.shmu.ac.ir/index.php/site/article/view/1543

مقدمه: افزايش سريع تعداد سالمندان در كشور مسأله نگران‌كننده‌اي براي تأمين نيازهاي اين گروه سني در زمينه‌هاي بهداشت، سلامت و رفاه آنان به حساب مي‌آيد. در اين ميان اقامت سالمندان در سراهاي سالمندان نيز پديده‌اي است كه رو به افزايش بوده و اثرات اين پديده بر سلامت جسمي و رواني سالمندان غيرقابل انكار است. هدف از اين مطالعه تعیین ارتباط افسردگی و کیفیت زندگی سالمندان مقیم و غیر‌مقیم سرای سالمندان مي‌باشد. مواد و روش‌ها: در مطالعه تحلیلی- مقطعي حاضر تعداد 315 نفر از سالمندان انتخاب شدند بدین صورت که80 نفر از ساکنان خانه سالمندان شهرستان شاهرود (39/25%) و همچنین 235 نفرسالمندان غیر‌مقیم (جامعه) (61/74%) به شیوه نمونه‌گیری غیرتصادفی در دسترس در سال 1393 انتخاب شدند. ابزار جمع‌آوری اطلاعات شامل پرسشنامه اطلاعات دموگرافیک، فرم کوتاه افسردگی سالمندان (GDS) جهت تعیین افسردگی و پرسشنامه کیفیت زندگی SF-36 (فرم کوتاه) برای مقیاس کیفیت زندگی بود که از طریق مصاحبه چهره به چهره با سالمندان تکمیل گردید. پس از تکمیل پرسشنامه‌ها، تجزیه و تحلیل داده‌ها با استفاده از نرم‌افزار SPSS نسخه 16 انجام گرفت. نتايج: میانگین سن سالمندان مورد پژوهش در گروه سالمندان مقیم سرای سالمندان (38/67) و غیر‌مقیم (17/66) بود. همچنین سطح نمره افسردگي هر دو گروه در حد متوسط قرارداشت. ارتباط معنادار و همبستگی مثبت بین ابعاد جسمی و روانی و نمره کل کیفیت زندگی با نمره افسردگی سالمندان مقیم سرای سالمندان وجود داشت (003/0=P) ولی در سالمندان غیرمقیم و همچنین در مجموع سالمندان ارتباط معنادار بین متغیرهای ذکرشده وجود نداشت (33/0=P). نتیجه‌گیری: با‌توجه به نتایج این مطالعه تأثیر منفی سکونت سالمندان در مراکز نگهداری بر بسیاری از جوانب زندگی این افراد مشخص شد و تشویق هر چه بیشتر خانواده‌های دارای سالمند به نگهداری آنان تحت حمایت خود به‌عنوان بهترین راه پیشگیری از بسیاری از مشکلاتی که ممکن است برای آنها رخ دهد، پیشنهاد می‌گردد.

Title: اعتباریابی نسخه فارسی پرسشنامه نگرانی ایالت پنسیلوانیا در بیماران مبتلا به مولتیپل اسکلروزیس

http://knh.shmu.ac.ir/index.php/site/article/view/1596

مقدمه: نگرانی یک سازه روان‌شناختی شایع در مولتیپل اسکلروزیس(MS) است که در مطالعات گذشته داده‌هاي اندكي از خواص روان‌سنجي ابزارهاي سنجش نگرانی در جمعيت بيماران MS وجود دارد. از اینرو پژوهش ‌حاضر با هدف اعتبارسنجی پرسشنامه نگراني ايالت پنسيلوانيا (PSWQ) که برای سنجش نگرانی ابزاری رایج است، در جمعيت بيماران ايراني مبتلا به MS انجام شد. مواد و روش‌ها: طرح پژوهش، مقطعي و از نوع اعتبارسنجی بود که در آن دو گروه از بیمارانMS  (80=n) و همتایان سالم آنها (80=n) به شیوه مبتني‌بر هدف نمونه‌گیری شده و از نظر عوامل جمعیت‌شناختی همتا گردیدند. جامعه هدف این مطالعه شامل تمامی بیماران عضو انجمن MS استان گیلان در سال 91-1390 بود. پایایی همسانی درونی، اعتبار سازه، همزمان، پیش‌بین و افتراقی PSWQ بررسی شده و آزمودنی‌ها از لحاظ علايم افسردگی، اضطراب، وسواس فکری- عملی و شاخص شدت کلی (GSI) در چک لیست نشانه‌های بیماری90 تجدیدنظر شده (SCL-90-R) و ویژگی نوروزگرایی در پرسشنامه شخصیتی نئو (NEO- FFI) ارزیابی شدند. نتایج: همسانی درونی PSWQ مناسب بود (74/0=α). ضریب همبستگی گویه- نمره‌کل برای عامل نگرانی عمومی مطلوب‌تر بوده و در دامنه 21/0 تا 60/0 قرار داشت. نتایج اعتبار همزمان نشان داد که PSWQ از رابطه معناداری با کلیه سازه‌های مرتبط با ابزارهای پژوهشی برخوردار بود (001/0≥P). به‌علاوه PSWQ نسبت معناداری از واریانس نمرات علايم آسیب‌شناسی روانی و نوروزگرایی را حتی پس از کنترل عوامل مخدوش‌کننده پیش‌بینی نمود. سرانجام، تحلیل تمایز نشان داد PSWQ می‌تواند دو گروه بیماران و همتایان سالم را از نظر نگرانی افتراق دهد و صحت طبقه‌بندی کلی آن 1/63 % می‌باشد. نتیجه‌گیری: PSWQ از خواص روان‌سنجی مطلوبی در جمعيت بيماران ايراني مبتلا‌به MS برخوردار مي‌باشد و می‌تواند در موقعیت‌های بالینی و پژوهشی مرتبط به‌کار رود.

Title: تأثير نوعی پروبیوتیک بر کلونیزه شدن روده‌ای و تهاجم بافتی سالمونلا انتریتیدیس در جوجه‌های گوشتی

http://knh.shmu.ac.ir/index.php/site/article/view/1608

مقدمه: سالمونلا انتریتیدیس یکی از مهم‌ترین عوامل بیماری‌های با منشاء غذایی در انسان است که اغلب از طریق مصرف فرآورده‌های آلوده پرندگان منتقل می‌شود. پروبیوتیک‌ها مکمل‌های غذایی حاوی میکروارگانسیم‌های زنده هستند که از طریق پدیده حذف رقابتی می‌توانند باعث کاهش آلودگی سالمونلایی شوند. این مطالعه با هدف بررسی اثر نوعی پروبیوتیک بر پایه باکتری‌های لاکتوباسیلوس، بیفیدوباکتریوم و انتروکوکوس بر کلونیزه شدن روده‌ای و تهاجم بافتی سالمونلا انتریتیدیس در جوجه‌های گوشتی انجام گرفت. مواد و روش‌ها: تعداد 128 جوجه یک روزه در یک طرح کاملاً تصادفی به چهار تیمار هر کدام با 4 تکرار 8 قطعه‌ای تقسیم شدند. تیمارهای 1 و 2 در طول آزمایش آب آشامیدنی حاوی 120 میلی‌گرم در لیتر پروبیوتیک را دریافت کردند، اما تیمارهای 3 و 4 آب معمولی استفاده کردند. در سن 2 روزگی، تیمارهای 1 و 3 با ورود جوجه‌هایی که یک روز قبل با cfu 105 × 5/1 سالمونلا تلقیح شده بودند (سیدر) مورد چالش قرار گرفتند. در سنین 3، 5، 7 و 21 روزگی، نمونه‌هایی از سکوم و کبد تهیه و از نظر آلودگی سالمونلایی بررسی شدند. نتایج: جوجه‌های تحت آزمایش هیچ‌گونه نشانه بالینی و تلفاتی نداشتند. سیدرها در طول آزمایش به سالمونلا آلوده بودند. کلونیزه شدن سالمونلا در سکوم به‌طور محسوس تحت تأثير پروبیوتیک قرار نگرفت، ولی جداسازی سالمونلا از نمونه‌های کبدی به‌طور نسبی 55 درصد کاهش یافت. نتیجه‌گیری: افزودن پروبیوتیک به آب جوجه‌های تازه به دنیا آمده از نظر درمانگاهی می‌تواند سودمند باشد، اما توجه به رعایت نکات بهداشتی در گله‌های مادر و نتاج آنها اهمیت بیشتری دارد.

Title: مقایسه عملکرد دو مدل‏ پیش‏بینی متاستاز براساس تکنیک‏های داده‏کاوی در بیماران سرطان پستان

http://knh.shmu.ac.ir/index.php/site/article/view/1613

مقدمه: با شناسایی فرآیند متاستاز و عوامل مؤثر بر آن به بهبود و بقای طولانی مدت بیماران کمک شایانی خواهد شد. هدف از مطالعه حاضر بررسی و شناسایی عوامل تأثیرگذار در پیش‏بینی متاستاز سرطان پستان با استفاده از ابزارهای داده‏کاوی است. داده‏كاوي ابزار کشف دانش از میان انبوهی از داده است كه امروزه در زمينه‏هاي مختلفي كاربرد پيدا كرده است. تشخيص بيماري‏ در علم پزشكي يكي از زمينه‏هاي رو به رشد و پركاربرد داده‏كاوي است. مواد و روش‌ها: در این پژوهش پس از آماده‏سازی داده‏ها، 2025 رکورد قابل استفاده مورد تجزيه و تحليل قرار گرفت. سپس با استفاده از الگوریتم‏های شبکه عصبی و CHAID به کشف الگوهایی که به پیش‏بینی متغیرهای تأثیرگذار بر متاستاز در بیمار کمک می‏کند، پرداخته‏ایم. نتایج: براساس نتایج متغیرهای Stage تومور، نوع عمل جراحی، نوع سرطان براساس پاتولوژی مهمترین متغیرهای پیش‏بینی‌کننده متاستاز هستند. نتیجه‌گیری: مقایسه عملکرد مدل‏ها در این پژوهش نشان می‏دهد که الگوریتم‏های CHAID و شبکه عصبی در پایگاه داده مورد استفاده، روش‏های مناسبی برای پیش‏بینی متاستاز در بیماران سرطان پستان می‏باشد.

Title: بهینه‌سازی رآکتور بیوفیلمی MBSBR براي کنترل نسبت غذا به ميكروارگانيسم و شوک آلی وارده به سیستم با تأکید بر حذف LAS از فاضلاب صنایع شوینده

http://knh.shmu.ac.ir/index.php/site/article/view/1642

مقدمه: در ميان روش‌هاي مختلف تصفيه، استفاده از فن‌آوری‌هاي مرتبط با فرآیندهاي بیولوژیکی از جمله سیستم‌های بیوفیلمی دارای اهميت ويژه‌اي مي‌باشد. این تحقیق جهت بهینه‌سازی رآکتور بیوفیلمی MBSBR (Moving bed sequenced batch reactor) در کنترل نسبت F/M و شوک آلی وارده به سیستم با تأکید بر حذفLAS  (Linear alkylbenzen sulfonate) از فاضلاب صنایع شوینده انجام شد. مواد و روش‌ها: در این بررسی از یک پایلوت پلکسی‌گلاس شفاف با حجم کلی 35 لیتر و حجم مفید 30 لیتر استفاده و در 3% پرشدگی مدیا (60، 50، 40%) راهبری گردید. بار آلی ورودی به سیستم به میزان 1000 mg/L و متغیرهای LAS به مقادیر 250 mg/L، 200، 100 بود. نتایج: نتایج حاصله از تحقیق حاکی از قابلیت بالای سیستم MBSBR در تصفیه فاضلاب صنایع شوینده بوده و حداکثر میزان راندمان به‌دست آمده از بار آلی و LAS در زمان ماند هیدرولیکی 5/2 ساعت با بار آلی ورودی به سیستم به میزان 1000 mg/L و LAS به میزان ورودی 100 mg/L (برای COD) و 200 mg/L (برای LAS) در نرخ پرشدگی 50% به‌ترتیب به مقدار 3/90 و 71/99 درصد به‌دست آمد. در شرایط بهینه محدوده‌ی نسبت F/M در 50% پرشدگی مدیا در محدوده‌ی (1/0-03/0) به‌دست آمد. همچنین راندمان حذف LAS با اعمال شوک کیفی در دو مقدار 1500 mg/L و 2000 به‌ترتيب به مقادیر 12/98 و 42/96 درصد رسید که حاکی از شوک‌پذیر بودن سیستم بیوفیلمی MBSBR می‌باشد. نتیجه‌گیری: نتایج این مطالعه نشان‌دهنده توانایی بالای راکتور MBSBR برای حذف LAS از فاضلاب با اعمال شوک‌های متفاوت است که می‌توان از آن به‌عنوان یک سیستم بیولوژیکی مؤثر در فرآیندهای تصفیه استفاده نمود.

Title: بررسی تأثیر آموزش خودمدیریتی برکیفیت زندگی بیماران دیابتی نوع دو

http://knh.shmu.ac.ir/index.php/site/article/view/1522

مقدمه: آموزش در دیابت یک مسأله بنیادی و مهم در دستیابی به کیفیت زندگی مطلوب است. یکی از مؤثرترین عوامل در کیفیت آموزش، انتخاب آموزش‌های مناسب است. این مطالعه با هدف تعیین تأثیر آموزش خودمدیریتی بر کیفیت زندگی بیماران دیابتی نوع دو انجام شد. مواد و روش‌ها: این مطالعه، یک کارآزمایی بالینی بود که بر روی 98 بیمار دیابتی نوع دو انجام شد که از طریق تخصیص تصادفی در دو گروه کنترل و مداخله قرار گرفتند. ابزار جمع‌آوری اطلاعات شامل پرسشنامه کلینیکی خلاصه شده کیفیت زندگی بیماران دیابتی (DQOL-BCI) به زبان فارسی، پرسشنامه دموگرافیک و مشخصات بیماری بود. قبل از شروع مداخله، پرسشنامه‌ها توسط بیماران تکمیل شد و سپس مداخله به‌صورت آموزش خودمدیریتی برای گروه مداخله به اجرا در آمد و بعد از دو ماه مجدداً پرسشنامه کیفیت زندگی تکمیل شد. برای تجزیه و تحلیل داده‌ها از نرم‌افزار آماری SPSS و آزمون‌های آماری مجذور کای، آزمون دقیق فیشر، تی زوجی و مستقل و سطح معنی‌داری 05/0 استفاده شد. نتايج: دو گروه از نظر متغیرهای دموگرافیک با یکدیگر همگن بودند. میانگین نمره کیفیت زندگی قبل از مداخله بین دو گروه تفاوت معنی‌دار نداشت (82/0=P) اما بعد از مداخله، میانگین نمره کیفیت زندگی بین دو گروه تفاوت معنی‌دار پیدا کرد (002/0=P). نتیجه‌گیری: نتایج مطالعه حاکی از بهبود کیفیت زندگی به‌دنبال آموزش خودمدیریتی بود. باتوجه به نقش مهم آموزش در مدیریت و کنترل بیماری‌های مزمن از جمله بیماری دیابت، استفاده از این نوع آموزش جهت دستیابی به کیفیت زندگی مطلوب توصیه می‌شود.

Title: الگوی تغییرات آسپارتات آمینوترانسفراز و آلکالین فسفاتاز در طول دوره کوفتگی عضلانی تأخيری ناشی از ورزش مقاومتی در پاسخ به نوعی مكمل‌سازي پروتئین وی در دانشجویان پسر کم تحرک

http://knh.shmu.ac.ir/index.php/site/article/view/1615

مقدمه: علیرغم ویژگی‌های آنتی‌اکسیدانی پروتئین وی، اثر آن روی آنزیم‌های کبدی متعاقب ورزش استقامتی در افراد ناورزیده به خوبی مشخص نشده است. مطالعه حاضر با هدف تعیین اثر مکمل‌سازی پروتئین وی بر آسپارتات آمینوترانسفراز (AST) و آلکالین فسفاتاز (ALP) تحت عنوان آنزیم‌های کبدی در طول کوفتگی تأخيری ناشی از ورزش مقاومتی انجام شد. مواد و روش‌ها: در این مطالعه کارآزمایی بالینی تصادفی دو سو كور، 28 دانشجوی پسر غیر ورزشکار (12±78 کیلوگرم) به شیوه تصادفی در دو گروه مکمل‌سازی پروتئین وی (4/0 گرم به ازای هر کیلوگرم وزن بدن در 3 روز متوالی) و دارونما تقسیم شدند. نمونه‌های خون در شرایط زمانی قبل، بلافاصله، 24، 48 و 72 ساعت پس از یک جلسه ورزش مقاومتی شدید در قالب انقباض‌های برونگرای پا با منظور اندازه‌گیری میزان فعالیت ALP و AST به‌عمل آمد. از آزمون تحلیل واریانس با اندازه‌گیری مکرر جهت مقایسه داده‌ها بین دو گروه استفاده شد. نتایج: در مقایسه با پیش آزمون، افزایش معنی‌داری در فعالیت AST و ALP بلافاصله پس از قطع آزمون در هر دو گروه مشاهده شد (05/0>P). بر پایه یافته‌های حاصل از آزمون تحلیل واریانس، مکمل‌سازی پروتئین وی به تغییر معنی‌داری در سطوح ALP و AST در دوره‌های تأخيری (24، 48 و 72 ریکاوری) نسبت‌به گروه دارونما منجر نشد (05/0>P).نتيجه‌گيري: باتوجه با یافته‌های حاضر، اینگونه نتیجه‌گیری می‌شود که مکمل‌سازی پروتئین وی بعد از ورزش مقاومتی شدید آنزیم‌های تعیین آسیب کبدی را در افراد ناورزیده متأثر نمی‌کند.

Title: اعتبارسنجی پروتکل پلکان فرانسیس به‌روش تجزيه و تحليل گازهای تنفسی و طراحی معادله بومی برآورد ظرفیت هوازی در پسران ایرانی

http://knh.shmu.ac.ir/index.php/site/article/view/1657

مقدمه: اندازه‌گیری دقیق، ایمن و ساده ظرفیت قلبی- تنفسی بچه‌ها در شرایط تحقیقاتی و بالینی اهمیت بالایی دارد. بنابراین، ارزیابی اعتبار آزمون‌های میدانی ورزشی خارج از محیط آزمایشگاهی قابل تأمل است. هدف این مطالعه سنجش اعتبار و پایایی پروتکل پلکان فرانسیس بر مبنای تجزيه و تحليل گازهای تنفسی (VO2,VCO2,RER) و طراحی معادله بومی برای برآورد ظرفیت هوازی پسران ایرانی است. مواد و روش‌ها: حداکثر اکسیژن مصرفی (VO2peak) به دو شیوه آزمون استاندارد بیشینه روی تریدمیل با سنجش مستقیم گازهای تنفسی و آزمون میدانی پلکان فرانسیس روی نیمکت الکترومکانیکی (ارگواستپ) با قابلیت تنظیم ارتفاع در 138 پسر سالم 8 تا 13 ساله اندازه‌گیری شد. برای ارزیابی اعتبار و پایایی پروتکل پلکان فرانسیس به‌ترتیب از ضریب همبستگی پیرسون و همبستگی درون طبقه‌ای درون گروهی (ICC) استفاده شد. نتایج: همبستگی معناداری بین حداکثر اکسیژن مصرفی در روش معیار و روش برآورد از طریق پروتکل پلکان فرانسیس مشاهده شد (001/0>P، 85/0=r). پایایی آزمون پلکان میدانی در حد عالی مشاهده شد (96/0=ICC). همچنين معادله خطی پیشگوی حداکثر اکسیژن مصرفی ویژه پسران ایرانی با استفاده از متغیرهای سن، BMI و ضربان قلب پایانی آزمون پله طراحی شد (001/0>P، 61/02=SEE، 88/0=r). نتیجه‌گیری: با‌توجه به اعتبار بالای آزمون پله فرانسیس متناسب با ابعاد آنتروپومتریکی، می‌توان از آن در برآورد سطح کارآیی دستگاه قلبی- تنفسی پسران (VO2peak) استفاده کرد. کاربست معادله بومی آزمون پله فرانسیس ویژه پسران ایرانی، می‌تواند در مراکز بالینی، ورزشی و در مطالعات علوم تندرستی قابل توجه باشد.

Title: تأثیر تمرین هوازی با مصرف ملاتونین بر مارکرهای آپوپتوزی HSP70 و HSP60 در قلب موش صحرایی نر پس از ایسکمی-ریپرفیوژن

http://knh.shmu.ac.ir/index.php/site/article/view/1680

مقدمه: هدف از اين تحقيق، بررسی تأثیر تمرین هوازی با مصرف ملاتونین بر بیان مارکرهای HSP70 و HSP60 میوکارد رت پس از ایسکمی ریپرفیوژن می‌باشد. مواد و روش‌ها: 30 رت صحرایی نر ویستار دو تا سه ماهه با وزن تقریبی 250-200 گرم مورد استفاده قرار گرفتند؛ موش‌ها به پنج گروه تقسيم شدند: گروه پایلوت (4n=)، كنترل (4n=)، ملاتونين (4n=)، هوازي (4n=) و هوازي ملاتونين (4n=). رت‌هاي گروه‌های ملاتونین به مدت یک ماه هر روز با دوز 10 میلی‌گرم به ازای هر کیلوگرم وزن بدن تیمار شدند و رت‌هاي گروه‌های تمرین هوازی و تمرین هوازی ملاتونین تحت دوره تمرین یک ماهه با تواتر تمرین سه جلسه در هفته روی تردمیل قرار گرفتند. سپس تمام رت‌ها در دو روز متوالی با فاصله 24 ساعت تحت تزریق داروی ایزوپرنالین با دوز 150 و 125 میلی‌گرم به ازای هر کیلوگرم وزن بدن قرار گرفتند؛ در نهایت رت‌ها پس از تأیید سکته تشریح شدند و اندازه‌گیری بیان ژن HSP70 و HSP60 به‌روشreal-time PCR  انجام شد. نتایج تحقیق با فرمول 2-∆∆ct و روش آماری تی مستقل، تحلیل واریانس یک‌طرفه و کروسکال والیس تحلیل شد. نتایج: نتايج مطالعه نشان داد كه میزان بیان ژن HSP70 در بین گروه‌های تجربی تفاوت معنی‌داری دارد (05/0P≥) ولي بین گروه ملاتونین و گروه هوازی ملاتونین تفاوت معنی‌داری وجود ندارد (989/0P≥). همچنين میزان بیان ژن HSP60 نيز در گروه‌های تحقیق تغییرات معنی‌داری نداشته است (297/0P≥). نتيجه‌گيري: تیمار ملاتونین و تمرین هوازی به تنهایی بر بیان ژن HSP70 و HSP60 تأثیر ناچیزی دارند اما تمرین هوازی با مصرف ملاتونین می‌تواند بیان ژن HSP70 و HSP60 را به ترتیب افزایش و کاهش دهد.
